# Conservative Management of Upper Tract Urothelial Carcinoma: A Narrative Review

**DOI:** 10.3390/jcm15093304

**Published:** 2026-04-26

**Authors:** Silvia Proietti, Cristian Axel Hernández-Gaytán, Federico De Leonardis, Stefano Gisone, Riccardo Scalia, Franco Gaboardi, Guido Giusti

**Affiliations:** San Raffaele Hospital, 20132 Milan, Italy; ahernandez.uro@gmail.com (C.A.H.-G.); deleonardis96@gmail.com (F.D.L.); stefanogisone@gmail.com (S.G.); scalia.ric@gmail.com (R.S.); gaboardi.franco@gmail.com (F.G.); drguidogiusti@gmail.com (G.G.)

**Keywords:** Upper Tract Urinary Carcinoma, endoscopic management, kidney-sparing surgery, laser technologies, intracavitary therapies

## Abstract

Upper tract urothelial carcinoma (UTUC) accounts for approximately 5–10% of urothelial malignancies and represents a clinically challenging disease due to its frequent presentation at advanced stages and its association with significant morbidity. Radical nephroureterectomy (RNU) with bladder cuff excision remains the standard treatment for high-risk disease; however, this approach inevitably results in loss of renal function and may significantly affect eligibility for cisplatin-based chemotherapy. In patients with imperative indications for renal preservation—including a solitary kidney, bilateral disease, or advanced chronic kidney disease—Kidney-Sparing Surgery (KSS) represents an essential therapeutic strategy. Technological advances in flexible ureteroscopy, improved visualization systems, and laser energy sources have significantly expanded the feasibility of conservative management. Ureteroscopic tumor ablation has become the cornerstone of KSS, allowing local disease control while preserving renal function. Although recurrence rates remain relatively high, repeated endoscopic treatment combined with strict surveillance protocols can achieve acceptable oncological outcomes in carefully selected patients. This narrative review summarizes the current evidence regarding conservative management of UTUC in imperative clinical situations, with particular emphasis on patient selection, endoscopic treatment modalities, laser technologies, economic implications, patient counselling, and follow-up strategies.

## 1. Introduction

Upper tract urothelial carcinoma (UTUC) represents a relatively uncommon malignancy arising from the urothelial lining of the renal pelvis and ureter, accounting for approximately 5–10% of all urothelial carcinomas [1,2]. Despite sharing histological characteristics with bladder cancer, UTUC demonstrates several distinct clinical features, including a higher incidence of invasive disease at diagnosis. Approximately 60% of UTUC tumors present as invasive lesions compared with 15–25% of bladder cancers [3].

Radical nephroureterectomy (RNU) with bladder cuff excision has historically been considered the gold standard treatment for localized UTUC, particularly for high-risk tumors [4]. However, radical surgery inevitably results in a reduction in renal function. This issue is particularly relevant because UTUC patients are typically elderly and frequently have multiple comorbidities such as hypertension, diabetes mellitus, and chronic kidney disease [5]. Furthermore, several studies have demonstrated that postoperative deterioration in renal function following RNU may render up to 50–60% of patients ineligible for cisplatin-based chemotherapy [6]. Given the increasing importance of systemic therapy in urothelial carcinoma management, preservation of renal function has become a critical therapeutic objective.

In this context, kidney-sparing surgery (KSS) has progressively gained attention. Advances in endoscopic technology—including digital flexible ureteroscopy, improved visualization systems, and modern laser platforms—have significantly expanded the feasibility of conservative treatment approaches [7].

KSS plays a particularly important role in patients with imperative indications, in whom radical nephroureterectomy would inevitably lead to dialysis. In such scenarios, preservation of renal function becomes a primary therapeutic objective, even when accepting a potentially higher risk of local recurrence.

## 2. Literature Search and Study Selection

This narrative review was based on a comprehensive literature search performed using PubMed, Scopus, and Web of Science databases. The search included studies published up to 2026 and used combinations of the following keywords: “*upper tract urothelial carcinoma*”, “*UTUC*”, “*kidney-sparing surgery*”, “*ureteroscopy*”, “*laser ablation*”, “*intracavitary therapy*”, and “*conservative management*”.

Relevant original articles, prospective and retrospective studies, systematic reviews, and meta-analyses were considered. Studies were selected based on their relevance to conservative management strategies for UTUC, including diagnostic approaches, endoscopic treatment, laser technologies, and adjuvant therapies. Articles not available in English, case reports, and studies lacking sufficient methodological detail were excluded. Additional references were identified through manual review of reference lists from selected articles.

## 3. Imperative Indications for Kidney-Sparing Treatment

Imperative indications for KSS arise when RNU would result in unacceptable functional consequences. These situations typically include patients with a solitary kidney, bilateral UTUC, or severe chronic kidney disease [8,9].

According to the current EAU guidelines, endoscopic management is recommended for low-risk tumors and selected high-risk tumors. Low-risk tumors include patients harboring a tumor size ≤2 cm, unifocal disease, low-grade cytology, low-grade on biopsy, and no evidence of invasive disease on CT-scan [4].

The rationale for conservative management in these patients is strongly supported by the poor prognosis associated with end-stage renal disease. Data from the United States Renal Data System demonstrate that five-year survival among patients undergoing chronic dialysis remains below 40% [10]. Cardiovascular complications represent the leading cause of mortality in this population [11].

In addition to reduced survival, dialysis significantly affects quality of life. Patients undergoing chronic hemodialysis frequently experience fatigue, reduced physical activity, and recurrent hospitalizations [12]. Consequently, preservation of renal function becomes a key therapeutic objective in selected patients with UTUC.

Clinical series evaluating conservative treatment in patients with imperative indications have reported encouraging outcomes, despite relatively high recurrence rates [13]. For instance, Proietti et al. conducted a retrospective study including 29 patients with imperative indications managed conservatively, encompassing a total of 137 endoscopic procedures. The authors reported an overall recurrence rate of 61.1%, with a 24-month recurrence-free survival (RFS) of 22.2% in patients with high-grade tumors [9]. Similarly, a long-term analysis of 60 high-risk patients undergoing conservative management (29 imperative patients) demonstrated comparable oncological outcomes between elective and imperative cohorts, with no significant differences in overall-survival (OS), RFS, and metastasis-free survival (MFS) at 5 years [14].

Overall, these data suggest that conservative management may be considered in carefully selected patients with imperative indications, including selected cases of high-grade disease, multifocal lesions, or increased tumor burden (e.g., larger or more extensive lesions). However, this approach should be applied within a strict risk-adapted framework. Although local recurrence rates remain relatively high, repeated endoscopic management combined with close surveillance often allows effective disease control while preserving renal function and avoiding long-term dialysis dependency [15].

## 4. Emerging Diagnostic Modalities for UTUC

The current gold standard for the diagnosis of UTUC is ureteroscopic biopsy; however, it is associated with several limitations, including the risks of bleeding, ureteral perforation, intravesical recurrence, and a suboptimal correlation with final pathology staging [16]. Non-invasive diagnostic tools, including urinary cytology and imaging modalities such as computed tomography urography (CTU) and magnetic resonance urography (MRU), may support the diagnostic process; nevertheless, these approaches have significant limitations. Selective upper tract (barbotage) urinary cytology may improve diagnostic yield; however, its overall sensitivity remains low, particularly for low-grade disease. Similarly, imaging techniques have limited ability to detect flat lesions and may not reliably distinguish between benign and malignant conditions [17].

In recent years, novel urine-based and peripheral blood-based biomarkers have emerged as promising non-invasive diagnostic tools for UTUC [18]. Circulating tumor cells (CTCs) have been associated with higher tumor grade, increased risk of recurrence, and metastatic progression [19,20]. In a prospective study, Zhang et al. evaluated urine-based RNA assay comprising eight genes, reporting a sensitivity of 90%, specificity of 88.9%, and an overall diagnostic accuracy of 88.9%, with consistent performance even in low-grade UTUC [21]. Additionally, DNA methylation assays (e.g., Epicheck) and fluorescence in situ hybridization (FISH)-based tests (e.g., UroVysion) have demonstrated encouraging diagnostic performance, with reported sensitivities of up to 83% and 93% and specificities of up to 81% and 91%, respectively [22,23]. Furthermore, a recent systematic review and meta-analysis by Xiao et al. demonstrated that urine biomarkers achieve high overall diagnostic accuracy, with pooled sensitivity and specificity of 0.86 and 0.94, respectively. Among the different biomarker categories, DNA methylation assays showed the most balanced performance (sensitivity 0.89, specificity 0.92), gene mutation assays achieved the highest specificity (0.97), and RNA-based assays demonstrated the highest sensitivity (0.97), albeit with significant heterogeneity and lower specificity [24].

## 5. Endoscopic Management of UTUC

Endoscopic management represents the cornerstone of conservative treatment for UTUC. Flexible ureteroscopy allows direct visualization of the upper urinary tract and enables tumor biopsy, mapping, and laser ablation within the same procedure [25]. Technological advancements in digital ureteroscopy have significantly improved visualization and maneuverability within the collecting system, facilitating more precise tumor ablation. In addition, novel imaging modalities such as photodynamic diagnosis (PDD) and confocal laser endomicroscopy (CLE) may further enhance diagnostic accuracy and treatment efficacy. Fluorescent photosensitizers, including 5-aminolevulinic acid and hexaminolevulinate, can be administered to improve tumor detection and visualization [26,27]. As a result, ureteroscopic treatment has become increasingly feasible even in anatomically complex locations [28].

Retrograde access via flexible ureteroscopy is generally the preferred technique for tumors located in the pelvicalyceal system because it is associated with a low complication rate and a reduced risk of tumor seeding compared with percutaneous approaches, a rare but recognized concern [29]. For distal ureteral tumors, rigid ureteroscopy is typically the preferred approach [30,31]. However, ureteroscopy has been associated with an increased risk of intravesical recurrence in some studies [32]. Percutaneous management may be considered in selected cases, particularly for large tumors located within the renal pelvis or when ureteroscopic access is technically challenging. Nevertheless, with the evolution of modern flexible ureteroscopes, the need for percutaneous approaches has significantly decreased and is now generally reserved for specific anatomical situations [33].

Recurrence rates following endoscopic treatment range from 40% to 70% in most series, largely due to the multifocal nature of UTUC [31]. In a meta-analysis performed by Kawada et al. upper tract recurrence rates ranged from 28 to 85% across different studies. Despite these relatively high recurrence rates, CSS and OS appear comparable between carefully selected patients undergoing KSS and those treated with RNU [34]. Table 1 summarizes the clinical and oncological outcomes reported in studies evaluating KSS for the management of UTUC.

## 6. Laser Technologies in Conservative UTUC Treatment

Laser energy plays a fundamental role in the endoscopic treatment of UTUC. The ideal laser platform should provide precise tissue ablation, adequate hemostasis, and limited depth of penetration to reduce the risk of ureteral perforation or delayed stenosis [45,46].

The Holmium:YAG (Ho:YAG) laser remains the most widely used energy source for UTUC ablation because of its versatility and controlled tissue penetration [47]. Its wavelength (2120 nm) and high peak power allow for effective tumor ablation, although its coagulative properties are relatively limited compared with newer technologies.

In recent years, thulium-based laser technologies have gained increasing attention. The thulium fiber laser (TFL) has emerged as a promising energy source in endourology. It has a wavelength of 1940 nm with low peak power (maximum 500 W), which offers excellent coagulation, although it may be associated with increased carbonization. Clinical evaluation of TFL for conservative UTUC treatment has demonstrated effective tumor vaporization, excellent hemostatic control, and low complication rates; however, these findings are primarily derived from retrospective studies with limited sample sizes and short follow-up [15,48].

Similarly, the pulsed thulium:YAG (p-Tm:YAG) laser operates at a wavelength close to the absorption peak of water (2013 nm), enabling efficient soft-tissue interaction and improved hemostatic properties compared with Ho:YAG systems [45,49]. Experimental and preclinical studies comparing Ho:YAG and Tm:YAG lasers have demonstrated differences in tissue interaction, with thulium lasers producing a wider coagulation zone and a shallower incision depth, which may reduce the risk of deep tissue injury during tumor ablation [50]. P-Tm:YAG platforms have been introduced with the aim of combining the cutting efficiency of Ho:YAG with the hemostatic advantages of thulium systems. Early clinical experience suggests that p-Tm:YAG may provide balanced cutting and coagulative properties during ureteroscopic tumor ablation [36,51]. Nevertheless, current clinical evidence for p-Tm:YAG in UTUC remains preliminary, with limited prospective data available.

Overall, modern endoscopic laser platforms—including Ho:YAG, and thulium-based technologies such as TFL and p-Tm:YAG—represent promising tools for the conservative management of UTUC. However, their role in UTUC management is still evolving, and further prospective, comparative studies are required to establish their long-term oncological outcomes and safety profiles.

## 7. Current Role of Distal and Segmental Ureterectomy

Advances in ureteroscopic technology and imaging modalities have significantly improved the evaluation and the ability to obtain tissue for histopathological diagnosis, thereby supporting staging and treatment decision-making. However, despite these improvements, accurate risk stratification remains challenging and endoscopic assessment may not always reliably predict final pathology.

RNU, although considered the standard treatment, is associated with non-negligible morbidity, with major complication rates reported in up to 18.2% of cases. Surgical alternatives, including segmental ureterectomy (SU) and distal ureterectomy (DU), have been proposed depending on tumor location [52]. Certainly, a key advantage of these approaches over endoscopic management is the ability to obtain definitive pathological staging and grading, while preserving renal function, which is particularly relevant in selected high-risk tumors. However, it should be noted that prospective randomized trials comparing SU or DU with RNU or endoscopic management are currently lacking [53].

In a large meta-analysis including 33,241 patients, Calvillo-Ramirez et al. found no significant difference in 5-year CSS, OS, or RFS between SU and RNU. Notably, better short-term renal function outcomes were observed in the SU group; however, this finding should be interpreted with caution, as patients undergoing RNU more frequently presented with higher-stage (≥T2) and high-grade tumors, whereas smaller and lower-risk tumors were more commonly treated with SU [54]. Similarly, Abrate et al. conducted a retrospective study including 177 patients aged ≥ 75 years and reported comparable 3-year OS between SU and RNU, with a significantly higher complication rate observed in the RNU group (34% vs. 7.4%) [55]. Regarding DU, Soria et al. performed a multicentric study including 450 patients and reported favorable oncological outcomes, with 5-year ipsilateral ureteral RFS of 82%, intravesical RFS of 49%, CSS of 89%, and OS of 72%, with no differences between low- and high-risk groups [56]. More recently, comparative studies evaluating robotic-assisted DU vs robotic RNU have demonstrated comparable oncological outcomes, including OS, RFS, and MFS, while showing improved long-term renal function preservation in patients undergoing DU [57,58].

## 8. Intracavitary Therapies

In patients previously treated with KSS, recurrence rates can be as high as 90%. Consequently, in addition to endoscopic tumor ablation, intracavitary therapies have emerged as an effective strategy for UTUC management. A meta-analysis performed by Soria and colleagues demonstrated a recurrence rate of 35% at 30 months following intracavitary therapy, with a 94% CSS [59]. Instillation of these therapies can be performed using three techniques: antegrade infusion via percutaneous access, reflux through a double-J stent, or retrograde administration via an open-ended ureteral catheter, with the latter reported as the most effective method [60,61].

For carcinoma in situ of the upper urinary tract (UT-CIS), additional intraluminal instillations with immunomodulators such as bacillus Calmette–Guérin (BCG) have demonstrated efficacy rates ranging from 63 to 100%, with a low complication rate [62]. Breda et al. evaluated adjuvant BCG instillation with histologically confirmed UT-CIS and reported a complete response in 58.8% of patients, with no significant difference in RFS compared to RNU or distal ureterectomy [38]. In another study, Fontanet et al. reported 5-year RFS and PFS rates of 49.6% and 72.3% respectively [63].

Adjuvant mitomycin C after therapeutic ureteroscopy for UTUC, of which 52% were low-grade tumors, was associated with a recurrence rate of 23.5% and a 7.7 fold lower risk of urothelial recurrence, with complication rates comparable to those of control group [41]. More recently, the development of reverse thermal gel formulations containing mitomycin has introduced a novel chemoablative strategy. The OLYMPUS trial evaluated UGN-101 in patients with low-grade UTUC and demonstrated a complete response rate of approximately 59% [40]. Similarly, in a multicenter study in which 84% of patients had low-grade tumors, percutaneous antegrade administration of mitomycin gel achieved a complete response in 59% of patients, while 38% had residual tumor [37]. This therapy has also been studied in patients with imperative indications and high-grade tumors, in whom 40% had no evidence of disease; among these patients, 88% maintained this status at a median of follow-up of 10.8 months [64]. However, this therapy may be associated with complications such as ureteral strictures in up to 9% of cases, highlighting the need for careful patient selection and close follow-up.

## 9. Economic Impact of Conservative Treatment

The economic implications of conservative management represent an important consideration in the treatment of UTUC. RNU performed in imperative situations may result in dialysis dependency, which carries substantial long-term healthcare costs. Economic analyses have demonstrated that preserving renal function through endoscopic management may lead to significant cost savings compared with long-term dialysis therapy. Even when repeated ureteroscopic procedures are required due to disease recurrence, the cumulative cost remains significantly lower than the long-term expenses associated with dialysis [65].

For patients with low-grade disease treated with RNU, the economic burden may be considerable, encompassing the initial surgical intervention, hospital readmissions, and long-term follow-up, especially in patients who become dialysis-dependent after the procedure [66]. From a cost-analysis perspective, KSS for UTUC may therefore represent a more cost-effective strategy. Pak and colleagues estimated that approximately $252,272 USD could be saved over a 5-year period when a patient undergoes KSS with recurrence management and surveillance compared with RNU followed by dialysis during the same timeframe [65].

## 10. Patient Counselling and Follow-Up Burden

Appropriate patient counselling represents a crucial component of conservative UTUC management, as the decision to perform a RNU may significantly affect quality of life (QoL) [67]. KSS should be viewed as a longitudinal management strategy rather than a single therapeutic intervention [68].

In a qualitative study performed by Hu et al., which evaluated patient experiences during the treatment of localized UTUC, it was found that patients frequently misunderstand the nature of the disease and experience pain or discomfort related to indwelling Foley catheters or ureteral stents [69]. Therefore, patients must be informed that repeated endoscopic procedures and strict surveillance protocols are often required to maintain disease control.

In imperative clinical situations, these discussions become particularly important because patients are often elderly, frail, and affected by multiple comorbidities [70]. Follow-up protocols typically include ureteroscopy, urinary cytology, and cross-sectional imaging. However, repeated ureteroscopic procedures may represent a significant burden for patients with limited functional reserve.

For this reason, individualized surveillance strategies may be considered. Imaging-based follow-up using CT urography or MR urography may help reduce the frequency of invasive procedures in selected patients, although ureteroscopy remains the most sensitive method for detecting small endoluminal recurrences.

## 11. Conclusions

KSS represents a valuable therapeutic option for patients with UTUC and imperative indications for renal preservation. Advances in endoscopic technology, laser systems, and intracavitary therapies have significantly expanded the feasibility of conservative treatment strategies.

Current evidence suggests that KSS provides the most favorable oncological outcomes in low-risk disease. In contrast, its role in high-risk UTUC is more limited and typically confined to selected patients with imperative indications, in whom preservation of renal function outweighs the increased risk of local recurrence.

Although recurrence rates remain relatively high, repeated endoscopic management combined with strict surveillance protocols may allow preservation of renal function without compromising CSS. Future research should focus on improving patient selection, optimizing follow-up strategies, and evaluating the long-term impact of emerging technologies in conservative UTUC treatment.

## Figures and Tables

**Table 1:** Studies evaluating Kidney-Sparing Surgery for the endoscopic management of localized UTUC.

Study (Year)	*n*	Indication	Laser Type/Technique	Adjuvant Treatment	Complication Rate	Oncologic Outcomes
Ye et al., 2025 [35]	33	High-risk UTUC (HER2+, imperative)	Tm:YAG	Disitamab vedotin + immunotherapy	9% UTI rate, no grade > 3 TRAEs	LRFS: 67% (1 yr), 64% (2 yr), OS 94%, CSS 100%, MFS 100%
Proietti et al., 2025 [36]	20	Low-grade 60%	Pulsed Tm:YAG	-	13.3% C-D grade I–II, no major complications	Residual tumor 20% (2nd look, all high-risk)
Rose et al., 2023 [37]	48	High-risk 79%, 100% imperative indication	Chemoablation (67% previous resection or ablation)	-	8% UTI (pyelonephritis), 8% ureteral stricture rate	40% CR; 88% maintained response
Proietti et al., 2022 [15]	28	Low-grade 67.8%	TFL	-	10.5% C-D grade I–II, one grade IIIB	Recurrence rate 21.7% and 17.7% at 6 and 12 mo, respectively
Territo et al., 2023 [38]	28	UT-CIS	None	-	17.6% C-D grade I–II	CR 58.8%; recurrence or persistence 41.2%. No difference vs. RNU in RFS/CSS
Bozzini et al., 2021 [39]	47	Low-grade 62.8%	Tm:YAG (continuous wave)	-	26.8% C-D grade I–II, 0% grade III	Recurrence rate: 19.2%
Kleinmann et al., 2020 [40]	71	Low-grade 100%	Chemoablation (52% previous ablation)	-	94% overall AEs, 37% serious events, 7% ureteral stricture rate	59% CR, 84% response rate at 12 mo
Gallioli et al., 2020 [41]	52	Low-grade 57%	Ho:YAG or TFL	Mitomycin vs. observation	16% C-D grade ≤ II, 4% grade III, 4.7% ureteral stricture rate	URFS: 28.8 vs. 18.8 mo; recurrence 23.5% vs. 55.5%
Proietti et al., 2020 [9]	29	Low-grade 37.9%	Ho:YAG or Tm:YAG	-	2.9% C-D grade III–IV	Recurrence rate 61.1%, OS 96.4%, RFS 31.7% (24 mo)
Wen et al., 2018 [42]	32	Low-grade 84%	TFL	-	No major complication rate/12.5% ureteral stricture rate	Recurrence rate 21.9%
Musi et al., 2018 [43]	42	Low-grade 69.1%	Tm:YAG (continuous wave) or TFL	-	85% C-D grade I–II	Recurrence rate 19%. No progression; 9.5% RNU. Median RFS 44 mo.
Cutress et al., 2012 [31]	73	Low-grade 72.6% (46.6% grade I, 26% grade II)	Nd:YAG/percutaneous resection	Mitomycin (24.7%)	19% overall complication rate, 16.4% ureteral stricture, 2.8% ≥ grade III	Recurrence rate 68.5%, CSS 88.9%, OS 69.7% (5 yr)
Krambeck et al., 2007 [8]	37	Low-grade 40.5% (5.4% grade I, 35.1% grade II)	KTP/Nd:YAG	BCG, mitomycin	Early: 21.6%; Late strictures: 13.5%	Recurrence rate 62%; 5-yr CSS: 49.3%
Martinez-Piñeiro., 1996 [44]	54	Low-grade 77.7% (44.4% grade I, 33.3% grade II)	Nd:YAG/electrofulguration/percutaneous resection	BCG, mitomycin, others	26% overall complication rate, mostly minor. 13% ureteral stricture rate	Recurrence rate 23.8%; CR 70%, CSM: 13.6%

Abbreviations: UT-CIS: Upper Tract Carcinoma in Situ; RNU: Radical Nephroureterectomy; RFS: Recurrence-Free Survival; OS: Overall Survival; CSS: Cancer-Specific Survival; CSM: Cancer-Specific Mortality; MFS: Metastasis-Free Survival; CR: Complete Response; TRAEs: Treatment-Related Adverse Events; C-D: Clavien-Dindo.

## Data Availability

No new data was generated.

## Abbreviations

The following abbreviations are used in this manuscript:

UTUC	Upper Tract Urothelial Carcinoma
RNU	Radical Nephroureterectomy
KSS	Kidney-Sparing Surgery
DU	Distal Ureterectomy
CLE	Confocal Laser Endomicroscopy
PDD	Photodynamic Diagnosis

## References

[1] Siegel R.L., Giaquinto A.N., Jemal A. (2024). Cancer statistics, 2024. CA Cancer J. Clin..

[2] Munoz J.J., Ellison L.M. (2000). Upper tract urothelial neoplasms: Incidence and survival during the last 2 decades. J. Urol..

[3] Holzbeierlein J., Bixler B.R., Buckley D.I., Chang S.S., Holmes R.S., James A.C., Kirkby E., McKiernan J.M., Schuckman A. (2024). Treatment of Non-Metastatic Muscle-Invasive Bladder Cancer: AUA/ASCO/SUO Guideline (2017; Amended 2020, 2024). J. Urol..

[4] Masson-Lecomte A., Baard J., Birtle A., Compérat E.M., Dominguez-Escrig J.L., Gontero P., Liedberg F., Mariappan P., Pradere B., Rai B.P. (2026). Upper Urinary Tract Urothelial Carcinoma.

[5] Seisen T., Colin P., Rouprêt M. (2015). Risk-adapted strategy for the kidney-sparing management of upper tract tumours. Nat. Rev. Urol..

[6] Lane B.R., Smith A.K., Larson B.T., Gong M.C., Campbell S.C., Raghavan D., Dreicer R., Hansel D.E., Stephenson A.J. (2010). Chronic kidney disease after nephroureterectomy for upper tract urothelial carcinoma and implications for the administration of perioperative chemotherapy. Cancer.

[7] Terrerito A., Foerster B., Shariat S., Roupret M., Gaya J., Palou J., Breda A. (2018). Diagnosis and kidney-sparing treatments for upper tract urothelial carcinoma: State of the art. Minerva Urol. Nefrol..

[8] Krambeck A.E., Thompson R.H., Lohse C.M., Patterson D.E., Elliott D.S., Blute M.L. (2007). Imperative Indications for Conservative Management of Upper Tract Transitional Cell Carcinoma. J. Urol..

[9] Proietti S., Marchioni M., Eisner B.H., Luciano R., Saitta G., Rodriguez-Socarras M.E., D’Orta C., Bellinzoni P., Schips L., Gaboardi F. (2021). Conservative treatment of upper urinary tract carcinoma in patients with imperative indications. Minerva Urol. Nephrol..

[10] Johansen K.L., Gilbertson D.T., Li S., Li S., Liu J., Roetker N.S., Hart A., Knapp C.D., Ku E., Powe N.R. (2025). US Renal Data System 2024 Annual Data Report: Epidemiology of Kidney Disease in the United States. Am. J. Kidney Dis..

[11] Go A.S., Chertow G.M., Fan D., Mcculloch C.E., Hsu C.-Y. (2004). Chronic Kidney Disease and the Risks of Death, Cardiovascular Events, and Hospitalization. N. Engl. J. Med..

[12] Raoofi S., Pashazadeh Kan F., Rafiei S., Hoseinipalangi Z., Rezaei S., Ahmadi S., Masoumi M., Mejareh Z.N., Benis M.R., Sharifi A. (2023). Hemodialysis and peritoneal dialysis—Health-related quality of life: Systematic review plus meta-analysis. BMJ Support. Palliat. Care.

[13] Campobasso D., Puliatti S., Micali S., Maestroni U.V. (2022). Conservative treatment of upper urinary tract urothelial carcinoma (UTUC) in patients with imperative indications: Not only an option. Minerva Urol. Nephrol..

[14] Baboudjian M., Territo A., Gallioli A., Verri P., Aumatell J., Izquierdo P., Uleri A., Tedde A., Basile G., Gaya J.M. (2023). Long-Term Oncologic Outcomes of Endoscopic Management of High-Risk Upper Tract Urothelial Carcinoma: The Fundació Puigvert’s Experience. J. Endourol..

[15] Proietti S., Johnston T., Pupulin M., Di Pietro S., Spagna S., Rico L., Lucianò R., Ventimiglia E., Villa L., Gaboardi F. (2022). Effectiveness and Safety of Thulium Fiber Laser in the Conservative Management of Patients with Upper Tract Urothelial Carcinoma. Eur. Urol. Open Sci..

[16] Subiela J.D., Territo A., Mercadé A., Balañà J., Aumatell J., Calderon J., Gallioli A., González-Padilla D.A., Gaya J.M., Palou J. (2020). Diagnostic accuracy of ureteroscopic biopsy in predicting stage and grade at final pathology in upper tract urothelial carcinoma: Systematic review and meta-analysis. Eur. J. Surg. Oncol..

[17] Leow J.J., Liu Z., Tan T.W., Lee Y.M., Yeo E.K., Chong Y.L. (2020). Optimal management of upper tract urothelial carcinoma: Current perspectives. Onco Targets Ther..

[18] Wei W., Fan P., Zhang Z., Wu D., Liu J., Wang L., Duan X., Zhang X., Ding D. (2024). A urine-based liquid biopsy for detection of upper tract urothelial carcinoma: A self-matched study. BMC Cancer.

[19] Zhang Z., Fan W., Deng Q., Tang S., Wang P., Xu P., Wang J., Yu M. (2017). The prognostic and diagnostic value of circulating tumor cells in bladder cancer and upper tract urothelial carcinoma: A meta-analysis of 30 published studies. Oncotarget.

[20] Shishido S.N., Ghoreifi A., Sayeed S., Courcoubetis G., Huang A., Ye B., Mrutyunjaya S., Gill I.S., Kuhn P., Mason J. (2022). Liquid Biopsy Landscape in Patients with Primary Upper Tract Urothelial Carcinoma. Cancers.

[21] Zhang H., Xu Y., Wang K., Zheng C., Li Y., Gong H., Liu C., Sheng M., Xu Q., Sun Y. (2024). Large-scale Prospective Validation Study of a Multiplex RNA Urine Test for Noninvasive Detection of Upper Tract Urothelial Carcinoma. Eur. Urol. Oncol..

[22] Territo A., Gallioli A., Diana P., Boissier R., Fontana M., Gaya J.M., Sanguedolce F., Calderón J., Piana A., Fontanet S. (2022). DNA Methylation Urine Biomarkers Test in the Diagnosis of Upper Tract Urothelial Carcinoma: Results from a Single-Center Prospective Clinical Trial. J. Urol..

[23] Pycha S., Trenti E., Mian C., Schwienbacher C., Hanspeter E., Palermo M., Pycha A., Danuser H., D’eLia C. (2023). Diagnostic value of Xpert^®^ BC Detection, Bladder Epicheck^®^, Urovysion^®^ FISH and cytology in the detection of upper urinary tract urothelial carcinoma. World J. Urol..

[24] Xiao H., Yi L., Ma Z., Dai K., Liu Y., Gao Y., Xu D., Huang H. (2026). Urine-Based Biomarkers in the Diagnosis of Upper Tract Urothelial Carcinoma: A Systematic Review and Meta-Analysis. J. Clin. Med..

[25] Grasso M. (2000). Ureteroscopic Management of Upper Urinary Tract Urothelial Malignancies. Rev. Urol..

[26] Yoshida T., Setsuda S., Ishizuka M., Inoue T., Kinoshita H., Matsuda T. (2020). Photodynamic Diagnosis with Oral 5-Aminolevulinic Acid for Upper Urinary Tract Carcinoma: A Prospective Clinical Trial. J. Endourol..

[27] Kata S.G., Aboumarzouk O.M., Zreik A., Somani B., Ahmad S., Nabi G., Buist R., Goodman C., Chlosta P., Golabek T. (2016). Photodynamic diagnostic ureterorenoscopy: A valuable tool in the detection of upper urinary tract tumour. Photodiagnosis Photodyn. Ther..

[28] Croghan S.M., Skolarikos A., Jack G.S., Manecksha R.P., Walsh M.T., O’Brien F.J., Davis N.F. (2023). Upper urinary tract pressures in endourology: A systematic review of range, variables and implications. BJU Int..

[29] Sorokin I., Welliver R.C., Elkadi O., Nazeer T., Kaufman R.P. (2013). Tumor Seeding of Percutaneous Nephrostomy Tract from Urothelial Carcinoma of the Kidney. Case Rep. Urol..

[30] Cornu J.N., Rouprêt M., Carpentier X., Geavlete B., de Medina S.G.D., Cussenot O., Traxer O. (2010). Oncologic control obtained after exclusive flexible ureteroscopic management of upper urinary tract urothelial cell carcinoma. World J. Urol..

[31] Cutress M.L., Stewart G.D., Wells-Cole S., Phipps S., Thomas B.G., Tolley D.A. (2012). Long-term endoscopic management of upper tract urothelial carcinoma: 20-year single-centre experience. BJU Int..

[32] Di Bello F., Gallioli A., Cannoletta D., Mancon S., Territo A., Diéguez L., Izquierdo P., Hernandez P., Sopena J.M.G., Palou J. (2026). Intravesical recurrence after therapeutic ureteroscopy for upper tract urothelial carcinoma: A meta-analysis. BJU Int..

[33] Giulioni C., Pirola G.M., Maggi M., Brocca C., Tramanzoli P., Stramucci S., Mantovan M., Perpepaj L., Cicconofri A., Gauhar V. (2024). Current Evidence on Utility, Outcomes, and Limitations of Endoscopic Laser Ablation for Localized Upper Urinary Tract Urothelial Carcinoma: Results from a Scoping Review. Eur. Urol. Open Sci..

[34] Kawada T., Laukhtina E., Quhal F., Yanagisawa T., Rajwa P., Pallauf M., von Deimling M., Bianchi A., Pradere B., Fajkovic H. (2023). Oncologic and Safety Outcomes for Endoscopic Surgery Versus Radical Nephroureterectomy for Upper Tract Urothelial Carcinoma: An Updated Systematic Review and Meta-analysis. Eur. Urol. Focus..

[35] Ye J., Chen Z., Liao X., Zhang S., Tu X., Wang Q., Zheng L., Chen K., Liao B., Zhang M. (2025). Endoscopic Thulium Laser Ablation with Disitamab Vedotin and Immunotherapy in High-risk Upper Tract Urothelial Carcinoma: A Prospective Pilot Study of a Novel Kidney-sparing Strategy. Eur. Urol. Oncol..

[36] Proietti S., De Leonardis F., Gaytan C.A.H., Monroy R.E., Gisone S., Scalia R., Gaboardi F., Giusti G. (2025). In medio stat virtus: Exploring the potential of the pulsed thulium:YAG laser in the endoscopic management of upper tract urothelial carcinoma. Cent. Eur. J. Urol..

[37] Rose K.M., Narang G., Rosen G., Labatte C., Dumitrascu C.I., Campagna J., Yu A., Manley B.J., Spiess P.E., Li R. (2023). Antegrade administration of mitomycin gel for upper tract urothelial carcinoma via percutaneous nephrostomy tube: A multi-institutional retrospective cohort study. BJU Int..

[38] Territo A., Fontanet S., Meneghetti I., Gallioli A., Sanguedolce F., Rodriguez-Faba Ó., Gaya J., Palou J., Huguet J., Breda A. (2023). Management of primary upper urinary tract carcinoma in situ diagnosed by ureteroscopic biopsy: Is bacillus Calmette-Guerin an alternative to nephroureterectomy?. Actas Urol. Esp..

[39] Bozzini G., Gastaldi C., Besana U., Calori A., Casellato S., Parma P., Pastore A., Macchi A., Breda A., Gozen A. (2021). Thulium-laser retrograde intra renal ablation of upper urinary tract transitional cell carcinoma: An ESUT Study. Minerva Urol. Nephrol..

[40] Kleinmann N., Matin S.F., Pierorazio P.M., Gore J.L., Shabsigh A., Hu B., Chamie K., Godoy G., Hubosky S., Rivera M. (2020). Primary chemoablation of low-grade upper tract urothelial carcinoma using UGN-101, a mitomycin-containing reverse thermal gel (OLYMPUS): An open-label, single-arm, phase 3 trial. Lancet Oncol..

[41] Gallioli A., Boissier R., Territo A., Vila Reyes H., Sanguedolce F., Gaya J.M., Regis F., Subiela J.D., Palou J., Breda A. (2020). Adjuvant Single-Dose Upper Urinary Tract Instillation of Mitomycin C After Therapeutic Ureteroscopy for Upper Tract Urothelial Carcinoma: A Single-Centre Prospective Non-Randomized Trial. J. Endourol..

[42] Wen J., Ji Z.G., Li H.Z. (2018). Treatment of upper tract urothelial carcinoma with ureteroscopy and thulium laser: A retrospective single center study. BMC Cancer.

[43] Musi G., Mistretta F.A., Marenghi C., Russo A., Catellani M., Nazzani S., Conti A., Luzzago S., Ferro M., Matei D.V. (2018). Thulium laser treatment of upper urinary tract carcinoma: A multi-institutional analysis of surgical and oncological outcomes. J. Endourol..

[44] Martinez-Pineiro J.A., Garch M.J., Martinez-Pineiro L. (1996). Endourological treatment of upper tract urothelial carcinomas: Analysis of a series of 59 tumors. J. Urol..

[45] Ortner G., Somani B.K., Güven S., Kitzbichler G., Traxer O., Giusti G., Proietti S., Liatsikos E., Kallidonis P., Ulvik Ø. (2023). Experts’ recommendations in laser use for the treatment of upper tract urothelial carcinoma: A comprehensive guide by the European Section of Uro-Technology (ESUT) and Training Research in Urological Surgery and Technology (T.R.U.S.T.) group. World J. Urol..

[46] Panthier F., Doizi S., Almeras C., Abid N., Traxer O. (2025). Laser applications in Endourology: Conservative treatment of upper tract urothelial carcinoma and ureteral strictures. Fr. J. Urol..

[47] Villa L., Haddad M., Capitanio U., Somani B.K., Cloutier J., Doizi S., Salonia A., Briganti A., Montorsi F., Traxer O. (2018). Which Patients with Upper Tract Urothelial Carcinoma Can be Safely Treated with Flexible Ureteroscopy with Holmium:YAG Laser Photoablation? Long-Term Results from a High Volume Institution. J. Urol..

[48] Solano C., Candela L., Panthier F., Corrales M., Traxer O. (2023). Initial experience with the graphical user interface for laser parameters setting of a new thulium fibre laser source device for urinary pathologies treatment. World J. Urol..

[49] Hernandez-Gaytan C.A., Proietti S., Leonardis FDe Mongelli L., Gaboardi F., Giusti G. (2025). Pulsed Thulium:YAG Laser for Lithotripsy. Eur. Urol. Focus.

[50] Proietti S., Rodríguez-Socarrás M.E., Eisner B.H., Lucianò R., Basulto Martinez M.J., Yeow Y., Rapallo I., Saitta G., Scarfò F., Gaboardi F. (2021). Thulium:Yag versus holmium:Yag laser effect on upper urinary tract soft tissue: Evidence from an ex vivo experimental study. J. Endourol..

[51] Scalia R., Gisone S., Escobar R., Proietti S., Gaboardi F., Giusti G. (2025). Pulsed TM-YAG laser (Thulio^®^): A new weapon in endourologists’ hand in the conservative management of imperative cases of Upper Tract Urothelial Carcinoma (UTUC). Int. Braz. J. Urol..

[52] Saini S., Deveshwar S.P., Hemal A.K. (2024). Narrative review of nephron-sparing surgical management of upper tract urothelial carcinoma: Is there a role for distal ureterectomy, segmental ureterectomy, and partial nephrectomy. Transl. Androl. Urol..

[53] Veccia A., Antonelli A., Checcucci E., Falagario U., Carrieri G., Guruli G., De Sio M., Simeone C., Porpiglia F., Autorino R. (2020). Segmental Ureterectomy for Upper Tract Urothelial Carcinoma: A Systematic Review and Meta-analysis of Comparative Studies. Clin. Genitourin. Cancer.

[54] Calvillo-Ramirez A., Chew L., Davis L., Casas-Huesca A.P., Esparza-Miranda L.A., Alvarado V.M.P., Cardenas J.S., Angulo-Lozano J.C., Macias-Cruz H.M., Robles-Aquije L. (2025). Oncologic and renal function outcomes of segmental ureterectomy vs. radical nephroureterectomy in upper tract urothelial carcinoma: A systematic review and meta-analysis. Urol. Oncol. Semin. Orig. Investig..

[55] Abrate A., Sessa F., Sessa M., Campi R., Sebastianelli A., Varca V., Pavone C., Vella M., Bartoletti R., Ficarra V. (2022). Segmental Ureterectomy Versus Radical Nephroureterectomy in Older Patients Treated for Upper Tract Urothelial Carcinoma. Clin. Genitourin. Cancer.

[56] Soria F., Liedberg F., Stahl E., Dominguez-Escrig J.L., Moschini M., Pradere B., D’aNdrea D., Teoh J.Y.-C., Capoun O., Soukup V. (2025). Perioperative and Oncological Outcomes of Distal Ureterectomy for Upper Tract Urothelial Carcinoma (UTUC): A Multicentre Study from the European Association of Urology Non–muscle-invasive Bladder Cancer/UTUC Guidelines Panels with a Focus on Survival Free from Ipsilateral UTUC Recurrence. Eur. Urol. Oncol..

[57] Ditonno F., Franco A., Veccia A., Bologna E., Wang L., Abdollah F., Finati M., Simone G., Tuderti G., Helstrom E. (2024). Robotic distal ureterectomy for high-risk distal ureteral urothelial carcinoma: A retrospective multicenter comparative analysis (roBUUST 2.0 collaborative group). Minerva Urol. Nephrol..

[58] Saini S., Lukas V., Pathak R.A., Hemal A.K. (2023). Robot-Assisted Laparoscopic Ureteral Reconstruction for Malignant Pathology: Single-Center Experience with Analysis of Perioperative, Functional, and Oncologic Outcomes. J. Endourol..

[59] Foerster B., D’Andrea D., Abufaraj M., Broenimann S., Karakiewicz P.I., Rouprêt M., Gontero P., Lerner S.P., Shariat S.F., Soria F. (2019). Endocavitary treatment for upper tract urothelial carcinoma: A meta-analysis of the current literature. Urol. Oncol. Semin. Orig. Investig..

[60] Pollard M.E., Levinson A.W., Shapiro E.Y., Cha D.Y., Small A.C., Mohamed N.E., Badani K.K., Gupta M. (2013). Comparison of 3 Upper Tract Anticarcinogenic Agent Delivery Techniques in an Ex Vivo Porcine Model. Urology.

[61] Khargi R., Connors C., Ricapito A., Yaghoubian A.J., Gallante B.E., Khusid J.A., Atallah W.M., Gupta M. (2023). Adjuvant intraluminal therapies for upper tract urothelial carcinoma. Transl. Androl. Urol..

[62] Tomisaki I., Kubo T., Minato A., Fujimoto N. (2018). Efficacy and Tolerability of Bacillus Calmette-Guérin Therapy as the First-Line Therapy for Upper Urinary Tract Carcinoma In Situ. Cancer Investig..

[63] Fontanet S., Gallioli A., Baboudjian M., Huguet J., Territo A., Gaya J.M., Gavrilov P., Izquierdo P., Verri P., Algaba F. (2023). Topical instillation of BCG immunotherapy for biopsy-proven primary upper urinary tract carcinoma in situ: A single institution series and systematic review. Urol. Oncol. Semin. Orig. Investig..

[64] Rose K.M., Murray K.S., Labbate C., Woldu S., Linehan J., Jacob J., Kaimakliotis H., Dickstein R., Feldman A., Matin S.F. (2023). Mitomycin Gel (UGN-101) as a Kidney-sparing Treatment for Upper Tract Urothelial Carcinoma in Patients with Imperative Indications and High-grade Disease. Eur. Urol. Focus..

[65] Pak R.W., Moskowitz E.J., Bagley D.H. (2009). What Is the Cost of Maintaining a Kidney in Upper-Tract Transitional-Cell Carcinoma? An Objective Analysis of Cost and Survival. J. Endourol..

[66] Thacker K., Raman J.D., McLean T., Said J., Oliver L., Gore J.L. (2021). Understanding the Economic Burden of Treating Low-Grade Upper Tract Urothelial Cancer in the United States. Urol. Pract..

[67] van Doeveren T., Remmers S., Atema V., van den Bergh R.C.N., Boevé E.R., Cornel E.B., van der Heijden A.G., Hendricksen K., Cauberg E.C., Jacobs R.A. (2024). Short-term Changes in Health-related Quality of Life of Patients Undergoing Radical Surgery for Upper Urinary Tract Urothelial Carcinoma: Results from a Prospective Phase 2 Clinical Trial. Eur. Urol. Open Sci..

[68] Figaroa O., Schuil H., Kamphuis G., van den Brink L., Hendriks N., Henderickx M., Bins A., van Moorselaar J., Ajayi L., Bus M. (2025). Challenges in Follow-up After Kidney-sparing Surgery for Upper Tract Urothelial Carcinoma: Insights from a Delphi Consensus. Eur. Urol. Oncol..

[69] Song S., Smith A., Hu B. (2023). The patient experience with localized upper tract urothelial cancer. Urol. Oncol. Semin. Orig. Investig..

[70] Filipenko D., Acquadro M., Ghatnekar O., Samuelsen C.H., Griebsch I. (2026). Exploring Patient Perspectives on Upper Tract Urothelial Cancer: A Qualitative Interview Study. Oncol. Ther..

